# Baseline geriatric 8 (G8) screening tool predicts overall survival in metastatic urothelial carcinoma treated with immune checkpoint inhibitors

**DOI:** 10.1093/oncolo/oyaf361

**Published:** 2025-12-24

**Authors:** Christopher H Rhee, Subir Goyal, Yuan Liu, Dylan J Martini, Julie M Shabto, Bassel Nazha, Omer Kucuk, Bradley C Carthon, Mehmet Asim Bilen, Jacqueline T Brown

**Affiliations:** Medical College of Georgia, Augusta University/University of Georgia Medical Partnership, Athens, GA, United States; Winship Cancer Institute of Emory University, Atlanta, GA, United States; Department of Biostatistics and Bioinformatics, Emory University, Atlanta, GA, United States; Winship Cancer Institute of Emory University, Atlanta, GA, United States; Department of Biostatistics and Bioinformatics, Emory University, Atlanta, GA, United States; Department of Medicine, Massachusetts General Hospital, Boston, MA, United States; Columbia University College of Physicians and Surgeons, New York, NY, United States; Piedmont Cancer Institute, Atlanta, GA, United States; Winship Cancer Institute of Emory University, Atlanta, GA, United States; Department of Hematology and Medical Oncology, Emory University School of Medicine, Atlanta, GA, United States; Winship Cancer Institute of Emory University, Atlanta, GA, United States; Department of Hematology and Medical Oncology, Emory University School of Medicine, Atlanta, GA, United States; Winship Cancer Institute of Emory University, Atlanta, GA, United States; Department of Hematology and Medical Oncology, Emory University School of Medicine, Atlanta, GA, United States; Winship Cancer Institute of Emory University, Atlanta, GA, United States; Department of Hematology and Medical Oncology, Emory University School of Medicine, Atlanta, GA, United States

**Keywords:** bladder cancer, urothelial carcinoma, geriatric oncology, immunotherapy, immune checkpoint inhibitor

## Abstract

**Background:**

Metastatic urothelial carcinoma (mUC) remains highly lethal despite advances in treatment. There is limited data surrounding the efficacy and toxicity of immune checkpoint inhibitors in treating elderly patients due to the high bar of clinical trial participation. The Geriatric 8 (G8) screening tool is a quick, 8-item questionnaire designed to assess frailty in older adults. This study evaluates the G8 tool’s utility in predicting overall survival (OS) and progression-free survival (PFS) in patients with mUC treated with immune checkpoint inhibitors.

**Materials and Methods:**

A retrospective analysis was conducted on 48 mUC patients treated with ICI monotherapy at Winship Cancer Institute between 2016 and 2019. Baseline G8 scores were calculated from clinical notes. Primary outcomes included OS and PFS, analyzed using Kaplan-Meier, Cox proportional hazards models, and multivariate analysis adjusted for demographic and clinical variables.

**Results:**

An impaired baseline G8 score predicted worse OS but not PFS. The incidence of immune-related adverse events was lower but not statistically significant in patients with impaired G8 scores (HR, 0.31; OR, 0.09-1.14; *P* = .077).

**Conclusion:**

The G8 screening tool effectively predicts OS in older mUC patients treated with ICIs, highlighting its potential utility in clinical decision-making. Frail patients, as identified by the G8, had significantly worse survival outcomes, underscoring the need for tailored therapeutic approaches in this vulnerable population. Further studies are warranted to validate these findings and explore interventions that may improve outcomes for frail, older patients with mUC.

Implications for Practice In older patients with metastatic urothelial carcinoma (mUC), accurately predicting overall survival is crucial for optimizing treatment strategies, given a lack of prospective data in this population specifically. This study demonstrated that the Geriatric 8 (G8) tool, a low-cost screening test for identifying frailty, correlated with overall survival in this cohort of older patients treated with immune checkpoint inhibitors. By incorporating the G8 score into clinical practice, oncologists may better identify older patients at higher risk for mortality, allowing them to better align treatment with patient goals and expectations.

## Introduction

Bladder cancer is the ninth most common cancer worldwide. Even when treated with state-of-the-art systemic therapy, the disease is deadly with a median survival is 31.5 months.^1^ Although immune checkpoint inhibitors (ICIs) have transformed urothelial carcinoma (mUC) management, older patients, who constitute the majority diagnosed with advanced mUC, are underrepresented in clinical trials, resulting in limited real-world data on efficacy and toxicity in older populations.

Comprehensive geriatric assessment (CGA) allows for the determination of physiologic age in older patients, a way of “staging the aging.” Domains assessed by CGA include functional status, fall risk, medical comorbidities, polypharmacy, cognition, psychological impairments like depression or anxiety, nutrition, and social support.^2^ These assessments are recommended by leading oncology organizations to help identify vulnerabilities not captured in routine oncologic evaluations but are often too time-consuming for routine clinical practice.^3-5^

The G8 geriatric screening tool is an 8-question screen that assesses food intake, weight loss, mobility, neuropsychological conditions, body mass index (BMI), concomitant medications, health status, and age and can suggest geriatric vulnerability or frailty with a sensitivity of 85% and specificity of 65%.^6^ The G8 has demonstrated prognostic value across multiple cancer types but has not yet been evaluated in older patients with mUC receiving ICI therapy. Although the standard-of-care first-line treatment in *most* patients with mUC is combination chemoimmunotherapy, many frail patients who are not chemotherapy candidates still receive single-agent ICIs in clinical practice today. This study evaluates the G8 as a prognostic biomarker in older mUC patients undergoing ICI therapy, aiming to enhance clinical decision-making for this vulnerable population.

## Methods

We performed a retrospective analysis of patients aged 70 and older with mUC treated with ICI (including anti-PD-1 or PD-L1 agents) in any line of treatment at Winship Cancer Institute between 2016 and 2019. Patients were identified through a pharmacy database.

Demographic and clinical data, including age, sex, race, BMI, Eastern Coorperate Oncology Group (ECOG) performance score, prior therapies, baseline laboratory data within 2 weeks of immunotherapy initiation, treatment response, and immune-related adverse events (irAEs), were collected from electronic medical records. A baseline G8 score was evaluated retrospectively using past clinic notes from physicians, advanced practice providers, social workers, and registered dieticians.

Progression-free survival (PFS) and overall survival (OS) were the primary outcome measures assessed from the start date of ICI until death or clinical or radiographic progression, respectively. Patients were censored at the last follow-up. Objective response rate (ORR) was also measured. Radiographic progression was defined by significant tumor growth on scans that led to treatment change; response was defined by tumor size decrease when noted by the radiologist on imaging reports. The univariate analysis (UVA) of each covariate with PFS and OS was tested by the Cox proportional hazards model with hazard ratio (HR) and its 95% confidence interval (CI) being reported. Logistic regression was performed in the UVA for ORR and irAEs. Multivariable analysis (MVA) was carried out by controlling for age, race, sex, smoking status, baseline BMI, number of prior lines of therapy, and number of metastatic sites. Multivariable analysis models were built using a backward variable elimination strategy, and only variables with *P* < .20 were retained in the final model. The overall significance level was set at *P* < .05. The relationship between G8 scores (≤14 impaired vs >14 non-impaired) and survival outcomes was calculated via Kaplan-Meier analyses.

## Results

### Baseline demographic and disease characteristics

A total of 48 patients were included. The median age was 73.5 years, and patients were mostly male (66.7%) and white (75%). A total of 70.8% of patients had a baseline G8, suggesting geriatric vulnerability despite 74.4% of patients being scored by their physician as having an ECOG performance score of 0-1.

### Baseline G8 score and survival outcomes in Kaplan-Meier analysis

The median follow-up for the entire cohort was 26.12 months (95% CI 9.97-32.24). The median overall survival (mOS) of the entire cohort was 9.9 months (95% CI 5.5-12.4). The median progression-free survival (mPFS) of the entire cohort was 2.7 months (95% CI 2.2-4).

**Figure 1.**
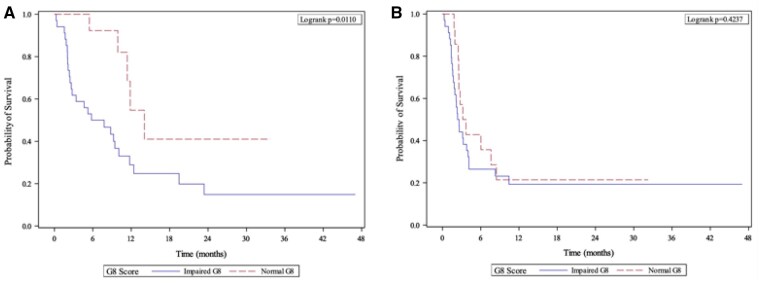
Kaplan-Meier estimates of (A) overall survival and (B) progression-free survival in patients with mUC treated with immunotherapy stratified by baseline G8 score.

An impaired G8 score at the start of treatment predicted a worse OS (Figure 1A). The mOS of the patients with impaired (≤14) and non-impaired (>14) G8 scores was 6.8 months (95% CI 2.4-10.1) versus 14 months (95% CI 9.9-not evaluable [NE]), respectively (*P *= .0110). A baseline impaired G8 score was not associated with a lower PFS (Figure 1B). The mPFS of the patients with impaired and non-impaired G8 scores was 2.5 months (95% CI 1.7-4) versus 3.4 months (95% CI 2.5-8.5), respectively (0.4237).

### Baseline G8 score and survival outcomes in univariate and multivariate analyses

The association baseline G8 score with survival outcomes, including PFS and OS, was assessed in both univariate and multivariate analyses (Table 1). In UVA, an impaired G8 score was significantly associated with decreased OS (HR, 3.01; CI, 12.9-8.45; *P *= .011). Progression-free survival (HR, 1.33; CI, 0.66-2.68; *P *= .424) and ORR (OR, 0.28; CI, 0.05-1.47; *P *= .131) were not significantly associated with an impaired G8 score.

**Table 1.** UVA and MVA analyses of the impact of baseline G8 score on survival outcomes and incidence of irAEs.

			OS		PFS		ORR		irAEs	
	**Variable**	**Score**	HR (CI)	*P*-value	HR (CI)	*P*-value	OR (CI)	*P*-value	OR (CI)	*P*-value
**UVA**	Baseline G8	Impaired	3.01 (1.29-8.45)	.011^a^	1.33 (0.66-2.68)	.424	0.28 (0.05-1.47)	.131	0.31 (0.09-1.14)	.077
		Normal (ref)	N/A	N/A	N/A	N/A	N/A	N/A	N/A	N/A
**MVA^†^**	Baseline G8	Impaired	3.59 (1.37-9.39)	.009^a^	1.49 (0.74-3.03)	.267	0.16 (0.02-1.17)	.072	0.31 (0.09-1.14)	.077
		Normal (ref)	N/A	N/A	N/A	N/A	N/A	N/A	N/A	N/A

† The MVA was built by controlling for age, race, sex, smoking status, baseline BMI, prior lines of therapy (#), and # of metastatic sites. Backward selection with an alpha level of removal of 0.2 was used;

a statistical significance at alpha < 0.05.

Abbreviations: BMI, body mass index; MVA, multivariable analysis; UVA, univariate analysis.

In MVA, an impaired G8 score was significantly associated with decreased OS (HR, 3.59; CI, 1.37-9.39; *P *= .009). However, PFS (HR, 1.49; CI, 0.74-3.03; *P *= .267) and ORR (OR, 0.16; CI, 0.02-1.17; *P *= .072) were not significantly associated with an impaired G8 score.

### Baseline G8 score and immune-related adverse events in univariate and multivariate analyses

The association baseline G8 score with incidence of irAEs was assessed in both univariate and multivariate analyses (Table 1). In both UVA and MVA, an impaired G8 score was nearly significantly associated with lower rates of irAEs (HR, 0.31; OR, 0.09-1.14; *P *= .077).

## Discussion

This exploration suggests that the G8 geriatric screening tool is a significant predictor of overall survival in older patients with mUC treated with ICIs, underscoring the value of incorporating geriatric screening into treatment decision-making for older adults. We found that an impaired G8 score at baseline, a biomarker suggestive of geriatric vulnerability, was a significant predictor of worse OS, but not necessarily worse PFS or ORR. This may suggest that our more frail or functionally vulnerable patients may not live as long despite cancer-directed care, *independent of their response to the drug*. Frail older patients may have diminished functional reserve and medical comorbidities competing with the cancer itself as the primary survival-limiting factor.^7^ Frailty may predict worse survival even when a cancer is responding to immunotherapy, which is extremely important in informing our clinical practice when taking care of these patients. Specifically, we need to have realistic expectations and communication skills to convey those expectations to our older and vulnerable patients so that they can choose a treatment or supportive care pathway most in line with their goals of care and values toward the end of their lives.

The rate of irAEs appeared lower in the impaired G8 group although it was not statistically significant. The efficacy of ICIs in cancer therapy hinges upon the presence of a robust immune response during treatment. Immunosenescence is defined by the gradual deterioration of the immune system that is brought on by natural aging. This is a multifaceted process, rendering older patients more susceptible to infections and diminishing their responsiveness to vaccinations.^8^ One hypothesis for this association could be a relationship between immunosenescence and diminished immune activation in response to ICIs among older patients. Another consideration is whether frailer patients with shorter survival have less time on treatment to experience an irAE.

The G8 score remains relevant even as first-line treatments evolve. Trials, such as EV-302/KEYNOTE-A39 and CheckMate-901, have established ICI-based combination therapies like pembrolizumab plus enfortumab vedotin and nivolumab plus gemcitabine-cisplatin as new standards of care.^1^^,^^9^ However, older patients were underrepresented in these studies due to strict eligibility criteria, limiting generalizability to real-world populations. This highlights the importance of assessing frailty, performance status, and functional reserve in older patients when considering ICI combinations.

Intensive treatment may carry burdens for older patients due to the concept of time toxicity. Examples of time toxicity may be seen in time spent on clinic visits, managing side effects, hospitalizations, and testing, relative to time gained.^10^ Patients too frail for chemotherapy still receive ICI monotherapy as investigated within this report and face many of the same burdens even with “lighter” treatment. Therefore, treatment decisions should be prefaced with a patient-centered discussion of values, preferences, and objectives. The G8 potentially offers a low-cost, practical tool to support risk-benefit discussions and shared decision-making in this population.

Although our findings support further study of the prognostic utility of the G8 score in mUC patients receiving ICI therapy, limitations include the small sample size (*n* = 48) and retrospective design, which may have introduced selection bias. Although we carefully applied the G8 retrospectively, some subjectivity is inherent, and there is potential for assessment bias. Our cohort received ICI monotherapy from 2016 to 2019, and thus, findings may not reflect outcomes with current combination regimens. This interval was selected in part because patients often received ICI monotherapy outside of the maintenance setting due to shifting FDA-approved indications for ICI use in mUC. Future research to refine our understanding of how to best treat our older patients with advanced UC is an essential complement to the therapeutic advances occurring in this ever-evolving field.

## Data Availability

The data underlying this article will be shared on reasonable request to the corresponding author.
